# An examination of substance use trends among adolescents receiving mental health treatment in Ontario

**DOI:** 10.3389/fpsyt.2025.1659388

**Published:** 2025-10-15

**Authors:** Shannon L. Stewart, Abbey L. Drew, Danielle Fearon

**Affiliations:** ^1^ Faculty of Education, Western University, London, ON, Canada; ^2^ Faculty of Health Sciences, Western University, London, ON, Canada

**Keywords:** adolescent substance use, risk factors, trauma, Child and Youth Mental Health Assessment, interRAI

## Abstract

**Introduction:**

Adolescent substance use continues to pose a significant public health concern due to its well-documented adverse effects on long-term health and well-being. Various risk factors, including mental health concerns (e.g., anxiety, depression), residential instability, prenatal exposure to substances, and various psychosocial concerns (e.g., low self-concept, poor social skills), have been recognized as contributors to adolescent substance use. Given the complex nature of substance use, it is essential to better our understanding of the factors that contribute to it.

**Methods:**

The current study aims to explore substance use trends among Ontario adolescents and examine the contexts in which these behaviors emerge. This study uses data from the interRAI Child and Youth Mental Health (ChYMH) assessment instrument, collected from youth receiving mental health services in Ontario between 2012 and 2022. Hierarchical logistic regression analysis was used to identify factors associated with triggering the Substance Use CAP.

**Results:**

In our sample, females, and older youth (15-18) were most likely to engage in substance use. Results indicated that residential instability, living alone or in a shelter, and living with a single parent are associated with substance use in adolescents. Furthermore, findings revealed that past or recent trauma, internalizing behavior, and school disengagement increased likelihood of engaging in substance use.

**Discussion and implications:**

This research provides researchers and clinicians with important insights into risk factors for substance use among adolescents which can be used to inform care planning and the development of prevention and early intervention efforts.

## 1 Introduction

Adolescent substance use remains a persistent public health problem due to its well-documented adverse effects on long-term health and well-being. In the United States, 78.2% of adolescents report having consumed alcohol, while 42.5% report using illicit drugs (1). In Canadian youth, rates of substance misuse have shown an upward trend, particularly after the COVID-19 pandemic. During the pandemic, elevated levels of stress, depression, and anxiety contributed to substance use rates being 50% higher than expected, with nearly 20% of youth engaging in weekly substance use (2). Adolescents who start using alcohol or drugs at an early age face a higher risk of several negative consequences. This developmental stage is crucial for biological, psychological, and social growth, making the brain especially sensitive to the long-term effects of substance use (3). Studies have also shown that marijuana onset at a young age is associated with reductions in cognitive functioning, memory, and processing speed (4, 5). Children who use alcohol or nicotine early, particularly in childhood or early adolescence, pose a higher risk of experiencing long-term substance dependence, lower levels of well-being in adulthood, reduced educational attainment and increased involvement in criminal activity (6).

A variety of intersecting risk factors contribute to substance use during adolescence. Mental health concerns such as depression, anxiety, self-harm, school disengagement and excessive screen time have all been positively associated with higher rates of substance use (6–8). In Ontario, Canada, studies have shown that adolescents experiencing residential instability or living with caregivers facing substance use disorder are particularly vulnerable (9). Almost half of adolescents facing residential instability in a Canadian sample study report multiple problematic substance use concerns (10). Additionally, prenatal exposure to substances such as cocaine has been shown to have a direct association with early-age onset marijuana use and heightened risk of long-term dependence (11). Psychosocial factors, including low self-concept, poor social skills, peer substance use, and impulsivity, have further been identified as predictors of adolescent substance misuse (12). Conversely, strong parent–adolescent relationships, greater parental monitoring, and increased time spent with family have demonstrated lower risk, contributing to decreased substance use and delinquency rates (4, 13).

Given the complex and multifaceted nature of adolescent substance use, understanding the risk factors of this issue is essential for formulating effective prevention and intervention strategies. This study utilizes data from the interRAI Child and Youth Mental Health (ChYMH) assessment instrument, collected from youth receiving mental health services in Ontario between 2012 and 2022. The research aims to explore substance use trends amongst Ontario adolescents and examine contexts in which these behaviors emerge. It was hypothesized that prenatal exposure to alcohol and drugs, early childhood trauma, residential instability, older age of the youth, and antisocial behavior would be powerfully associated with substance use, underscoring the importance of addressing these factors in clinical care planning and service delivery.

## 2 Methods

### 2.1 Sample

Data for this study included youth aged 12 to 18 years assessed in community (N = 11,592) or residential/inpatient (N = 925) mental health agencies in Ontario, Canada using the interRAI Child and Youth Mental Health Assessment (interRAI ChYMH; 14) instrument between January 1, 2012 and October 31, 2022. Data from the full completed assessments of all youth were included for further analyses.

### 2.2 Instrument

The interRAI ChYMH (14) is a semi-structured interview-based assessment tool that collects over 400 items used for identification information, treatment planning, indicators of mental health, sociodemographic, and clinical indicators. The instrument was designed for use with children and youth to assess their mental health and physical needs and identify areas of risk. Assessments are completed by trained clinicians overseeing care of the individual. Information is gathered from various sources such as interviews with the child/youth, family members, service providers, educators, observations, clinical records and case notes, and through consultation with other professionals (14). As a standard of practice, all agencies obtain consent from the families and child/youth. Embedded in the ChYMH are validated algorithms (Collaborative Action Plans (CAPs)) that can be triggered to prioritize needs and inform evidence-based care planning (15–18). The standardized assessment system has been applied across multiple contexts, such as supporting triaging, resource allocation, prioritization, and evaluation. The instrument is data-driven, and the embedded scales and algorithms have robust psychometric properties, and internal consistency (19–26). Data collected from agencies utilizing the ChYMH are entered into a deidentified web-based software system held in the interRAI Canada server. Ethics approval through Western University’s Ethics Board has been approved for secondary data analysis of data used in the present study. This study examined selected scales and CAPs embedded in the ChYMH.

### 2.3 Embedded CAPs

#### 2.3.1 Substance Use CAP

The Substance Use CAP (27) is a case finding tool used to identify youth using alcohol, illicit drugs, or misusing over-the-counter or prescribed medication. When triggered, the Substance Use CAP flags concerns related to the youth’s use and provides guidelines to eliminate use and manage side effects (28). The Substance Use CAP is based on the youth reporting recent use of a substance. The Substance Use CAP is triggered when youth report having done at least one of the following: Consumed alcohol to the point of intoxication at least once in the last 30 days, intentionally misused prescription or over-the-counter medication within the last 90 days, or used any other substances (i.e., inhalants, hallucinogens, cocaine or crack, stimulants, opiates (including synthetics) or cannabis) at any time.

#### 2.3.2 Traumatic Life Events CAP

The Traumatic Life Events CAP (29) can be triggered at two levels, and identifies individuals with immediate safety concerns (due to recent trauma), or those who are not in immediate danger, but have experienced prior traumatic events (14, 30, 31). Major life stressors include: serious accident or physical impairment, death or loss of a parent or primary caregiver, death or loss of other close family member, failing or dropping out of an educational program, immigration (including refugee status), living in a war zone or area of conflict, witnessing a severe accident, and victimization (i.e., crime, sexual, physical).

### 2.4 Embedded scales

The *Hyperactive/Distraction Scale* is a four-item scale assessing the frequency of impulsivity, hyperactivity, ease of distraction, and disorganization (19). Each item is rated based on a scale from 0 (not present) to 4 (exhibited daily in the last 3 days, 3 or more episodes or continuously), with a total score range of 0 to 16. The Scale was divided into four categories reflecting level of hyperactivity or distraction including low (scores ranging from 0 to 8), moderate (scores 9 to 10), high (scores 11 to 12), and very high (scores 13 to 16), respectively.

The *Parenting Strengths Scale* is a six-item scale reflecting the degree of strengths that the parent is demonstrating in parenting activities. The scale reflects items of ability to communicate effectively with the youth, assisting in the regulation of emotions, appropriate disciplinary approaches, providing warmth and support, appropriate supervision, and appropriate limit setting or expectations (18). The Parenting Strengths Scale (32) total score ranges from 0 to 12, with higher scores indicating lower levels of parenting strengths. The Scale was categorized into three levels including high strengths (scores ranging from 0 to 5), moderate strengths (scores from 6 to 8), and low strengths (scores 9 to 12).

The *School Disengagement Scale* is an eight-item scale, measuring elements of behavioral, emotional and cognitive disengagement. The scale includes items reflecting the presence of increased lateness or absenteeism, poor productivity or disruptiveness at school, conflict with school staff, current removal from school due to disruptive behavior, strong persistent dissatisfaction with school, current refusal to attend school, expressing intent to quit school, or poor overall academic performance (33). The School Disengagement Scale total scores range from 0 to 8, with higher scores indicating heightened disengagement. The Scale was categorized into low disengagement (scores from 0 to 3), moderate disengagement (scores from 4 to 5), and high disengagement (scores from 6 to 8).

The *Internalizing Scale* (measuring the frequency and severity of internalizing symptoms (20) and the Externalizing Scale (measuring the frequency of externalizing symptoms) both consist of 12 items that range from 0 to 48, with higher scores indicating greater symptoms. Items are scored from 0 (not present) to 4 (exhibited daily in the last 3 days, 3 or more episodes or continuously) to create a composite value. The *Externalizing Scale* includes items of both reactive (e.g., impulsivity, physical abuse, defiant behavior, argumentativeness), and proactive (e.g., stealing, bullying, preoccupation with violence or violent ideation, intimidation and threats of violence) behaviors (34). The Internalizing Scale includes factors of anxiety, anhedonia, and depression (20).

The *Risk of Harm to Others Scale* is a composite measure of violent ideation, threatened violence, violence to others, verbally abusive behavior, and socially inappropriate/disruptive behavior (28). The scale ranges from 0 (lowest) to 6 (highest), with higher scores indicating heightened risk of harm to others. The scale provides a valuable decision-support tool used to identify youth with increased likelihood of harming others (35).

### 2.5 Sociodemographic variables

Demographic variables such as age, sex (male, female or other), and primary language (English or other) were included. Age was categorized into 12 to 14 years old, 15 to 16 years old, and 17 to 18 years old. In the ChYMH assessment form, living arrangement includes alone, with parent(s) or primary caregiver(s), with sibling(s) (i.e., no parent(s)/primary caregiver(s), with relative(s), with foster family, or with nonrelative(s) (excluding foster family). Residential instability was ascertained based on responses of “yes” to having had 3 or more moves, no permanent address, homelessness, living in a shelter or “couch surfing” in the last two years. Additional living status indicators were measured based on residence at time of assessment, with responses of “yes” to living in a group home or shelter.

Additional social characteristics were also included such as the marital status of the parents, including never married, married, with a partner or significant other, widowed, separated, divorced, or marital status unknown. Maternal patterns of substance use during pregnancy, with response items of no, yes, or unknown/uncertain for both alcohol and drug use were also included. History of care, reflecting severe failure to provide basic needs for the child was included based on the youth’s age at earliest occurrence (none, 0 to 4 years, 5 to 11 years, or 12 to 18 years). History of care included three items including emotional neglect, physical needs, and safety needs. Positive endorsement to the youth having a peer group including individuals with persistent anti-social behavior was used to ascertain anti-social peer group.

### 2.6 Analytic approach

Pearson’s chi-square tests were used to investigate baseline sociodemographic or clinical characteristics with whether the youth triggered the Substance Use CAP. Factors identified as significant based on bivariate analyses, were then used for additional modelling. Hierarchical logistic regression analysis was used to identify factors associated with triggering the Substance Use CAP. Odds ratios and associated 95% confidence intervals were reported.

## 3 Results

Baseline demographic and clinical characteristics of the overall sample population are presented in **Table 1**. The overall sample was predominantly female (n=7,246) relative to other genders (males, n=5,173; other=98). The average age of youth that triggered the Substance Use CAP was 15.6 (SD: 1.7). Of youth who triggered the Substance Use CAP, more experienced residential instability in the last two years (19.3%), and had anti-social peer groups (34.0%) relative to those that did not trigger the Substance Use CAP. Most of the youth that triggered the Substance Use CAP lived with their parent(s) or primary caregiver(s) (81.4%, n=3,110), and less than half of parents were married (36.6%, n=1,399).

**Table 1:** Sample characteristics of youth in the ChYMH, stratified by the Substance Use CAP.

Description	Substance Use CAP				P value
	Not triggered (n=8,695)		Triggered (n=3,822)		
	%	N	%	N	
Age					<.0001
12-14	57.0	4,953	22.6	862	
15-16	32.4	2,821	52.4	2,002	
17-18	10.6	921	25.1	958	
Sex					0.036
Male	42.1	3,657	39.7	1,516	
Female	57.1	4,968	59.6	2,278	
Other	0.8	70	0.7	28	
Primary language					<.0001
English	94.9	8,251	96.8	3,699	
Other	5.1	444	3.2	123	
Living arrangement					<.0001
Alone	0.9	79	2.3	86	
With parent(s) or primary caregiver(s)	92.3	8,030	81.4	3,110	
With sibling(s), no parent(s)/primary caregiver(s)	0.3	27	0.6	25	
With other relative(s)	2.8	241	4.7	178	
With foster family	1.7	146	3.1	119	
With nonrelative(s), excluding foster family	2.0	172	7.9	304	
Residential instability					<.0001
Yes	5.7	496	19.3	737	
No/other	94.3	8,199	80.7	3,085	
Marital status of parents					<.0001
Never married	14.7	1,275	20.9	800	
Married	46.3	4,030	36.6	1,399	
Partner/significant other	1.6	137	1.8	69	
Widowed	2.6	226	2.0	78	
Separated	11.5	1,001	11.9	455	
Divorced	17.9	1,554	19.0	727	
Marital status unknown/other	5.4	472	7.7	294	
Group home					<.0001
Yes	3.7	320	11.0	420	
No	96.3	8,375	89.0	3,402	
Shelter					<.0001
Yes	1.0	88	5.0	192	
No	99.0	8,607	95.0	3,630	
Maternal alcohol consumption during pregnancy					<.0001
Yes	4.1	357	6.7	256	
No	68.0	5,916	59.2	2,263	
Unknown	27.9	2,422	34.1	1,303	
Maternal drug use during pregnancy					<.0001
Yes	3.5	305	6.4	245	
No	68.7	5,972	58.8	2,248	
Unknown	27.8	2,418	34.8	1,329	
Anti-Social Peer Group					
Yes	6.1	534	34.0	1,301	
No	93.9	8,161	66.0	2,521	
History of care includes severe failure to provide basic needs (youth’s age at earliest occurrence):					
Emotional neglect					<.0001
None	87.8	7,633	77.8	2,973	
0–4 years	7.7	672	12.5	479	
5–11 years	3.3	287	6.5	249	
12–18 years	1.2	103	3.2	121	
Physical needs					<.0001
None	92.4	8,031	86.0	3,288	
0–4 years	5.6	488	8.7	333	
5–11 years	1.6	139	3.6	138	
12–18 years	0.4	37	1.7	63	
Safety needs					<.0001
None	90.9	7,905	83.3	3,183	
0–4 years	6.1	527	9.4	360	
5–11 years	2.4	210	5.4	208	
12–18 years	0.6	53	1.9	71	

**Table 2** presents selected scales, CAPs and clinical characteristics of the sample population. Bivariate analyses suggested that of youth who triggered the Substance Use CAP, more experienced traumatic life events, with 43.5% having experienced prior trauma, and 21.4% having experienced immediate safety concerns, relative to those who rather than that did not trigger the Substance Use CAP. Youth that triggered the Substance Use CAP also reported more moderate (45.0%) to high (13.3%) risk of harm to others, and more moderate (16.9%) to high (6.3%) school disengagement relative to youth that did not trigger the Substance Use CAP.

**Table 2:** Selected collaborative action plans, scales and clinical characteristics of the sample population, stratified by the Substance Use CAP.

Description	Substance Use CAP				P value
	Not triggered (n=8,695)		Triggered (n=3,822)		
	%	N	%	N	
Traumatic Life Events CAP					<.0001
not triggered/unknown	48.3	4,198	35.1	1,340	
triggered *(Prior trauma)*	39.2	3,405	43.5	1,664	
triggered *(Immediate safety concerns)*	12.6	1,092	21.4	818	
Hyperactive/Distraction Scale (HDS)					<.0001
low (scores: 0-8)	75.1	6,530	70.1	2,680	
moderate (scores: 9-10)	8.3	720	10.8	412	
high (scores: 11-12)	7.9	686	9.0	345	
very high (scores: 13-16)	8.7	759	10.1	385	
Parenting Strengths Scale (PSS)					0.0004
high (scores: 0-5)	98.7	8,583	97.8	3,738	
moderate (scores: 6-8)	0.9	78	1.7	64	
low (scores: 9-12)	0.4	34	0.5	20	
School Disengagement Scale (SDS)					<.0001
low (scores: 0-3)	87.2	7,580	76.8	2,937	
moderate (scores: 4-5)	10.4	903	16.9	644	
high (scores: 6-8)	2.4	212	6.3	241	
Risk of Harm to Others Scale					<.0001
low/none	60.9	5,299	41.7	1,595	
moderate (scores 1-3)	31.8	2,766	45.0	1,720	
high (scores >=4)	7.3	630	13.3	507	
Internalizing Scale					<.0001
low/none (scores <=12)	61.8	5,373	54.4	2,080	
moderate (scores 13-15)	36.5	3,176	42.1	1,610	
high (scores >=36)	1.7	146	3.5	132	
Externalizing Scale					<.0001
low/none (scores <=12)	78.8	6,849	65.0	2,486	
moderate (scores 13-15)	20.3	1,766	33.3	1,271	
high (scores >=36)	0.9	80	1.7	65	

As shown in **Table 3**, 28.2% of youth who triggered the Substance Use CAP had consumed alcohol to the point of intoxication within the last 30 days. Within the last three days to a year, substances such as cannabis (91.3%), hallucinogens (14.8%), cocaine (12.0%), and stimulants (10.6%) were commonly used.

**Table 3:** Time since substance use among individuals that triggered the Substance Use CAP.

Description	Triggered (n=3,822)	
	%	N
Alcohol		
Number of days in the last 30 days consumed alcohol to the point of intoxication		
none	71.8	2,744
Daily - 9 or more days	28.2	1,078
Inhalants		
never	97.3	3,719
in the last 3 days - more than a year ago	2.7	103
Hallucinogens		
never	85.2	3,257
in the last 3 days - more than a year ago	14.8	565
Cocaine		
never	88.0	3,363
in the last 3 days - more than a year ago	12.0	459
Stimulants		
never	89.4	3,417
in the last 3 days - more than a year ago	10.6	405
Opiates		
never	93.4	3,568
in the last 3 days - more than a year ago	6.6	254
Cannabis		
never	8.7	331
in the last 3 days - more than a year ago	91.3	3,491

**Table 4** shows the adjusted estimates of factors associated with predicting the Substance Use CAP. Odds of triggering the CAP were highest for youth aged 17 to 18 (OR = 7.1, [6.2-8.1], <.0001), and youth aged 15 to 16 (OR = 4.4, [4.0-4.9], <.0001). The likelihood of triggering the Substance Use CAP was also heightened for youth reporting anti-social peer groups (OR = 5.8, [5.1-6.6], <.0001). Living alone, experiencing residential instability, and living in a shelter all showed significant positive associations with triggering the Substance Use CAP. Maternal substance use during pregnancy, and school disengagement also showed positive associations. Among other covariates in the model, additional associations were found. Odds of triggering the Substance Use CAP was relatively high for youth that triggered the Traumatic Life Events CAP (both for prior or recent trauma), youth with high internalizing behavior, and heightened disengagement from school.

**Table 4:** Results of the final logistic regression model examining factors associated with triggering the Substance Use CAP.

Characteristic	Final Model		P value
	c statistic: 0.803		
	OR	95% CI	
Age (reference: 12-14)			
15-16	**4.4**	**(3.96-4.88)**	**<.0001**
17-18	**7.1**	**(6.20-8.11)**	**<.0001**
Sex (reference: male)			
female	**1.3**	**(1.17-1.43)**	**<.0001**
other	1.3	(0.82-2.16)	0.250
Language (reference: English)			
other	**0.7**	**(0.55-0.87)**	**0.002**
Living arrangement (reference: with parent(s) or primary caregiver(s))			
Alone	**1.6**	**(1.06-2.32)**	**0.026**
With sibling(s), no parent(s)/primary caregiver(s)	1.2	(0.64-2.23)	0.583
With other relative(s)	1.2	(0.91-1.48)	0.229
With foster family	0.9	(0.67-1.26)	0.594
With nonrelative(s), excluding foster family	1.3	(0.99-1.68)	0.065
Residential Instability (reference: no/other)			
Yes	**1.7**	**(1.40-1.97)**	**<.0001**
Marital status of parents (reference: married)			
Never married	**1.5**	**(1.33-1.74)**	**<.0001**
Partner/significant other	1.3	(0.95-1.91)	0.093
Widowed	0.8	(0.58-1.08)	0.139
Separated	**1.3**	**(1.10-1.49)**	**0.001**
Divorced	**1.2**	**(1.07-1.37)**	**0.003**
Marital status unknown/other	1.1	(0.92-1.38)	0.265
Group home (reference: no)			
Yes	1.1	(0.89-1.38)	0.347
Shelter (reference: no)			
Yes	**1.5**	**(1.05-2.04)**	**0.024**
Maternal substance use during pregnancy (alcohol) (reference: no)			
Yes	0.8	(0.62-1.11)	0.203
Unknown	0.8	(0.53-1.06)	0.107
Maternal substance use during pregnancy (drug use) (reference: no)			
Yes	**1.9**	**(1.42-2.55)**	**<.0001**
Unknown	**1.7**	**(1.19-2.39)**	**0.003**
Anti-social Peer Group (peer groups includes individuals with persistent antisocial behavior) (reference: no)			
Yes	**5.8**	**(5.07-6.57)**	**<.0001**
History of care includes severe failure to provide for basic needs (youth's age at earliest occurrence):			
Emotional neglect (reference: none)			
0–4 years	1.1	(0.81-1.37)	0.699
5–11 years	1.2	(0.88-1.50)	0.310
12–18 years	1.3	(0.95-1.90)	0.098
Physical needs (reference: none)			
0–4 years	0.9	(0.63-1.27)	0.517
5–11 years	1.1	(0.74-1.61)	0.669
12–18 years	1.0	(0.59-1.88)	0.892
Safety needs (reference: none)			
0–4 years	0.9	(0.63-1.21)	0.411
5–11 years	1.1	(0.80-1.54)	0.531
12–18 years	1.1	(0.65-1.80)	0.750
Additional Scales and CAPs			
Traumatic Life Events CAP (reference: not triggered/unknown)			
triggered *(Prior trauma)*	**1.2**	**(1.12-1.39)**	**<.0001**
triggered *(Immediate safety concerns)*	**1.4**	**(1.19-1.58)**	**<.0001**
Risk of Harm to Others Scale (reference: low/none)			
moderate (scores 1-3)	**1.6**	**(1.43-1.78)**	**<.0001**
high (scores >=4)	**1.4**	**(1.12-1.65)**	**0.002**
Internalizing Scale (reference: low/none)			
moderate (scores 13-35)	1.0	(0.94-1.15)	0.421
high (scores >=36)	**1.5**	**(1.12-2.02)**	**0.007**
Externalizing Scale (reference: low/none)			
moderate (scores 13-35)	**1.3**	**(1.14-1.50)**	**0.0001**
high (scores >=36)	1.2	(0.76-1.88)	0.435
Hyperactive/Distraction Scale (HDS) (reference: low, scores 0-8)			
moderate (scores: 9-10)	1.1	(0.92-1.26)	0.325
high (scores: 11-12)	0.9	(0.73-1.03)	0.094
very high (scores: 13-16)	0.9	(0.72-1.02)	0.069
Parenting Strengths Scale (PSS) (reference: high strengths, scores 0-5)			
moderate strengths (scores: 6-8)	1.0	(0.70-1.59)	0.964
low strengths (scores: 9-12)	0.8	(0.38-1.53)	0.448
School Disengagement Scale (SDS) (reference: low, scores 0-3)			
moderate disengagement (scores: 4-5)	**1.3**	**(1.16-1.52)**	**<.0001**
high disengagement (scores: 6-8)	**1.6**	**(1.27-2.05)**	**<.0001**

Bolded values indicate statistically significant associations.

## 4 Discussion

This study used data from a large sample of mental health treatment-seeking children and youth to explore substance use trends amongst Ontario adolescents and examine contexts in which these behaviors emerge. In our sample, adolescents who triggered the Substance Use CAP were predominantly female. This finding may be reflective of the higher proportion of females in our sample and may not be reflect gender-based differences of substance use behavior in the general population. Existing research on the effects of gender on adolescent substance use produces mixed findings. While a substantial body of evidence indicates that substance use disorders are most prevalent in male adolescents (36, 37), recent studies show increasing rates of the disorder among females (38). These increases may be explained by neurobiological sex differences, growing mental health challenges linked to increased social media usage (e.g., self-esteem and body image issues), and gender-specific peer and family influences (39–41). Furthermore, evidence suggests an increased use of specific substances, such as psychostimulants and opioids, among females, which may be contributing to the observed rise in substance use within this group (40, 42). Emerging research also indicates that gender minority youth (i.e., non-binary, transgender, gender nonconforming youth) may be at heightened risk of developing substance use problems (43, 44). However, given the small sample of gender minority youth in our sample, meaningful comparisons could not be conducted.

With respect to age, the average age of adolescents that triggered the Substance Use CAP in our sample was 15.6 with the highest odds observed in youth ages 17-18. This is consistent with existing literature which reports that adolescent substance use disorders are most common among older youth (9, 45; 46). A higher prevalence of substance use disorders in older adolescents may be explained by increased access to substances. Moreover, factors such as an increase in life stressors and responsibilities may contribute to this finding.

The current study used hierarchical logistic regression to predict likelihood of triggering the Substance Use CAP. We found that experiencing residential instability was associated with triggering the Substance Use CAP. Similarly, our results indicate that living alone and living in a shelter are positively associated with substance use. This confirms existing findings as previous Canadian studies have indicated that adolescents facing residential instability present with multiple substance use concerns (9, 10). Adolescents living alone may experience substance use problems due, in part, to increased responsibilities, feelings of loneliness, and/or increased life stressors. Most participants in our sample live with a parent(s). However, among those who triggered the Substance Use CAP, less than half of their parents were married, suggesting that there may be an association between adolescent substance use disorder and parental marital status. Specifically, extant literature has indicated that those youth in two-parent families are at a reduced risk for substance use, including illicit drug use and delinquency (47–49). This is likely due to closer monitoring as well as decreased exposure to delinquent, substance-using peers (49).

Notably, results from the current study indicated that reporting anti-social peer groups was associated with triggering the Substance Use CAP. This finding adds insight to results from previous studies which indicate that poor social skills and peer pressure are associated with youth substance use (12, 50). Youth substance use in anti-social peer groups may be a consequence of poor family relations (51), feelings of loneliness and depression, potentially arising from low social engagement and increased time spent alone (52). Additionally, evidence suggests that concurrent use of substances, or adolescent polysubstance use, is associated with adverse mental health and behavioral outcomes which could further contribute to school disengagement (53, 54).

This study found that maternal drug use during pregnancy was associated with substance use. A study by Richardson et al. (11) reported similar findings indicating that prenatal exposure to substances such as cocaine increased early use of marijuana and dependence on illicit substances. Similar studies have confirmed these results demonstrating that prenatal exposure to various substances (e.g., alcohol, marijuana, cocaine, stimulants) increases risk of substance use disorders in children and adolescents, in addition to other disorders (e.g., FASD) (55, 56).

Finally, the study revealed that the odds of triggering the substance use CAP were significantly higher among youth that triggered the Traumatic Life Events CAP, reported high internalizing behavior, and reported high levels of school disengagement. These findings support the existing literature on adolescent substance use which indicates that trauma (57) and internalizing behaviors (58) are associated with an increase in youth substance use. Adolescents may utilize substances to cope with harmful effects associated with trauma and manage internalizing behavior. Consistent with our findings, previous studies have found associations between adolescent substance use and school disengagement. Some studies suggest that school disengagement may increase risk of substance use (33, 59), whereas other studies indicate that school disengagement may follow from increased substance use (60, 61). These findings suggest a potential bidirectional relationship between school disengagement and substance use in adolescents; however, further research is needed to confirm this relationship. Moreover, recent evidence suggests that fostering school engagement may serve as a protective factor against adolescent substance use, highlighting promising avenues for intervention (62).

### 4.1 Limitations and future directions

While there are several strengths, there are some limitations with this study that should be considered. First, as this study used cross-sectional data, claims about causation cannot be made. As a result, only potential risk factors for substance use in youth can be discussed. Future research investigating substance use in children and youth should consider utilizing longitudinal data so that causal relationships can be drawn. Second, substance use may be underreported due to fears of stigma, repercussions, and/or recall bias. Third, although data was collected from 2012 to 2022, this study did not specifically analyze changes in substance use during the COVID-19 pandemic. Research on the prevalence of substance use among adolescents during the pandemic produces mixed findings. Several studies report reduced substance use rates due to reduced social interactions and access to substances, while other studies indicate an increase in substance use as a coping mechanism for mental health challenges (63–65). Future research should consider exploring how risk factors for substance use among adolescents may have been impacted by the COVID-19 pandemic.

Additionally, while this study controlled for numerous variables and scales embedded in the ChYMH (e.g., age, sex, living status, internalizing and externalizing symptoms), it did not include additional factors associated with substance use such as socioeconomic status (SES) and co-occurring mental health diagnoses (9, 66). Low SES has been found to be associated with residential instability, antisocial peer groups, and maternal substance use which were identified as predictors of substance use in our study (67–69). Similarly, co-occurring mental health diagnoses (e.g., ADHD, depression, conduct disorder) have been found to increase risk of substance use (70). Future studies should consider collecting data on participants’ SES and co-occurring mental health conditions, and controlling for these factors in their analyses, to account for their effects on adolescent substance use.

Finally, although this study utilized a large and comprehensive sample of treatment-seeking children and youth, it focused on youth in Ontario. Non-clinical populations and children in regions outside of Ontario may differ in demographics, healthcare systems, service delivery models, and socioeconomic conditions. As such, findings may not be generalizable to non-treatment seeking youth or to children in other Canadian provinces or geographical regions outside of Canada. Future studies should examine clinical populations in other regions of Canada and the world to assess the consistency of these findings in diverse cultural, social, and health care contexts.

### 4.2 Implications for clinical practice

This study provides researchers and clinicians with valuable insights into the risk factors and patterns associated with adolescent substance use in Ontario. These findings can help guide the development of targeted interventions aimed at preventing substance use and promoting early intervention among adolescents. A comprehensive assessment tool such as the interRAI ChYMH can help clinicians and other mental health professionals understand adolescent’s strengths and needs, providing a solid foundation for effective care planning. Given that substance use was most prevalent among female adolescents (38, 71), clinicians should consider potential sex differences when assessing and providing recommendations for clients. Research suggests that neurobiological differences affecting reward processing regions in the brain may explain sex differences in substance use patterns (40). Clinicians with an understanding of biological sex differences can help identify at-risk populations and deliver targeted and effective care.

Findings from this study revealed that substance use is strongly associated with family consultation, underscoring the importance of taking family structure into account when developing policies and programs targeting youth substance use prevention (49). Poor family relations often are associated with greater residential instability (9);, trauma, and unmet physical and safety needs (57). These risk factors then increase the likelihood that youth will be more likely to develop anti-social peer groups that often contribute to school disengagement. It is important for clinicians to consider the factors that increase adolescents’ vulnerability to substance use to help prevent use and intervene at the earliest possible stage. Clinicians should consider using the interRAI ChYMH, which is a standardized and evidence-based assessment tool that effectively identifies populations at risk for problematic behaviors including substance use. When the ChYMH identifies youth at-risk for substance use, the Substance Use CAP is triggered. The Substance Use CAP is an effective evidence-based care planning tool that provides clinicians with best practices and recommendations for decreasing substance use in at-risk and currently using adolescents.

The various risk factors for adolescent substance use identified in this study highlight the need for an integrated, standardized assessment-to-intervention approach to support high-risk children and youth across multiple service sectors. Coordination across settings including schools, mental health agencies, hospitals, and youth justice facilities is needed to prevent vulnerable youth from falling through cracks between service sectors (28). Professionals across service sectors should consider utilizing the interRAI suite of instruments, which improves coordination across sectors to support identification of needs, and facilitate triaging and prioritization. By using evidence-based decisions, the interRAI suite enhances care planning and helps ensure consistent, coordinated support for at-risk youth (14).

### 4.3 Conclusions

Our study revealed that residential instability, living alone or in a shelter, and living at home with a single parent are associated with substance use in adolescents. Moreover, findings from our study revealed that past or recent trauma, emotional neglect, unmet physical and safety needs, internalizing behaviors, and school disengagement increased adolescents’ likelihood of engaging in substance use. Anti-social peer groups and prenatal exposure to substances were also found to increase risk of substance use in children and youth. In our sample, older youth (ages 17-18) were more likely to engage in substance use than younger children, and females were more likely to use substances than other genders. These findings provide researchers and clinicians with important insights into risk factors for substance use among adolescents which can be used to inform care planning and the development of prevention and early intervention efforts. This study highlights the importance of recognizing adolescents’ unique strengths and needs to guide effective treatment and intervention across numerous service sectors.

## Data Availability

The datasets for this article are not publicly available due to interRAI licensing and data sharing agreements. Requests to access these datasets should be directed to AD, adrew6@uwo.ca.
